# Decreased Emotional Dysregulation Following Multi-Modal Motion-Assisted Memory Desensitization and Reconsolidation Therapy (3MDR): Identifying Possible Driving Factors in Remediation of Treatment-Resistant PTSD

**DOI:** 10.3390/ijerph182212243

**Published:** 2021-11-22

**Authors:** Emily Tang, Chelsea Jones, Lorraine Smith-MacDonald, Matthew R. G. Brown, Eric H. G. J. M. Vermetten, Suzette Brémault-Phillips

**Affiliations:** 1 Department of Occupational Therapy, Faculty of Rehabilitation Medicine, University of Alberta, Edmonton, AB T6G 2G4, Canada; ewtang@ualberta.ca (E.T.); smithmac@ualberta.ca (L.S.-M.); 2 Heroes in Mind, Advocacy and Research Consortium (HiMARC), Faculty of Rehabilitation Medicine, University of Alberta, Edmonton, AB T6G 2G4, Canada; cweiman@ualberta.ca (C.J.); mbrown2@ualberta.ca (M.R.G.B.); 3 Neuroscience and Mental Health Institute (NMHI), University of Alberta, Edmonton, AB T6G 2E1, Canada; 4 Alberta Health Services, Edmonton, AB T5E 5R8, Canada; 5 Leiden University Medical Center, 2333 ZA Leiden, The Netherlands; e.vermetten@lumc.nl; 6 Department of Computing Science, University of Alberta, Edmonton, AB T6G 2E8, Canada; 7 ARQ National Psychotrauma Center, 1112 XE Diemen, The Netherlands; 8 Military Mental Health, Dutch Ministry of Defense, 3584 EZ Utrecht, The Netherlands

**Keywords:** 3MDR, treatment-resistant PTSD, military, veterans, mental health, emotional regulation

## Abstract

Multi-modal motion-assisted memory desensitization and reconsolidation therapy (3MDR), an interactive, virtual reality-assisted, exposure-based intervention for PTSD, has shown promising results for treatment-resistant posttraumatic stress disorder (TR-PTSD) among military members (MMs) and veterans in randomized controlled trials (RCT). Previous research has suggested that emotional regulation (ER) and emotional dysregulation (ED) may be factors which are correlated with symptom severity and maintenance of TR-PTSD. This embedded mixed-methods pilot study (*n* = 9) sought to explore the impact of 3MDR on ER and ED of MMs and veterans. Difficulties in Emotional Regulation Scale (DERS-18) data were collected at baseline, prior to each session, and at one week, one month, and three months postintervention and analyzed. Qualitative data collected from sessions, debriefs, and follow-up interviews were transcribed and descriptively analyzed. Results demonstrated statistically significant decreases in DERS-18 scores from preintervention to postintervention at each timepoint. Qualitatively, participants perceived improvements in ER within specified DERS-18 domains. We describe how 3MDR’s unique and novel approach addresses ED through cognitive–motor stimulation, narration, divergent thinking, reappraisal of aversive stimuli, dual-task processing, and reconsolidation of traumatic memories. More studies are needed to better understand the underlying neurobiological mechanisms by which 3MDR addresses ER and PTSD.

## 1. Introduction

Posttraumatic stress disorder (PTSD), one of the leading mental health disorders reported by military organizations worldwide, has detrimental effects, such as attrition and overburdening of healthcare resources [1,2]. Military members (MMs) are vulnerable to developing PTSD due to training and deployment experiences, and the stressful and often traumatic nature of their roles [3,4]. Symptoms of PTSD among MMs often include hypervigilance, reduced cognitive abilities, emotional dysregulation (ED), and comorbidities of anxiety and mood disorders [5]. MMs may also experience moral injury (MI) concurrently with PTSD, which is associated with feelings of shame, guilt, and distress following exposure to potentially morally injurious events (PMIEs) and violations of core morals and beliefs [6,7].

Treatment approaches for PTSD among MMs and veterans have variable effectiveness. Veterans see significantly worse treatment outcomes when compared with other populations that experience PTSD [6]. Further, conventional treatment dropout rates for veterans are as high as 78%, with approximately 65% of patients continuing to meet the threshold for PTSD diagnosis following treatment [8,9,10]. Conventional evidence-based PTSD therapies, such as cognitive behavioral therapy (CBT) and exposure therapies, are also less effective in the treatment of combat-related treatment resistant PTSD (TR-PTSD) subtype [10,11]. Given the potential severity of TR-PTSD symptoms and altering or limiting consequences, it is essential that MMs and veterans are offered treatments that are evidence-based and effective.

### 1.1. 3MDR Intervention

Multi-modal motion-assisted memory desensitization and reprocessing therapy (3MDR) is a novel interactive psychotherapeutic approach for TR-PTSD [8,9,10] that is delivered in a virtual reality environment. The intervention was initially developed in the Netherlands to treat MMs and veterans experiencing TR-PTSD who had limited success with other forms of treatment. Combining aspects of conventional PTSD therapies, 3MDR includes eye movement desensitization and reprocessing therapy (EMDR), virtual reality exposure therapy (VRET), and trauma-focused CBT [8,9,10,12], and introduces components such as motor engagement via treadmill walking. Data from recently published RCTs [10,13] demonstrate the efficacy of 3MDR therapy among MMs and veteran populations who experience TR-PTSD [14]. 

The 3MDR intervention used in this study consisted of eight sessions over an eight-week period, beginning with one to two pre-platform preparatory session(s), during which the patient selects and orders images and music. Symbolic representations in the form of images (i.e., photographs, sketches) related to the patient’s traumatic experiences are selected and ordered from least to most distressing. Music is also identified that reminds the patient of the past time of trauma, facilitates the emotional memory network (i.e., a piece of music that is activating of the trauma), and supports a return to the present (i.e., a second, contemporary piece that is soothing, compassionate, and joyful). Participants were also familiarized with the virtual environment and 3MDR protocol in a preparatory session. The six platform sessions were 90 min therapy sessions in the virtual reality environment (VRE), including a 30 min debrief. Each platform session involves three phases (pre-platform, platform, and post-platform). In the pre-platform phase, the therapist and patient confirm the order of images and music for the session. During the platform phase, the patient dons a safety harness and is accompanied by a 3MDR therapist while walking continuously on a treadmill at a self-selected pace. The patient first warms up by walking on the treadmill while listening to self-selected music connecting them to traumatic experiences and then, during each of seven 3–5 min cycles, walks down a virtual “hallway” toward a self-selected trauma-related image. The patient describes the image, physical sensations, and feelings to the therapist. The therapist assists the patient with generating descriptive words/phrases that are then projected in front of the image on the screen and which the patient is instructed to read aloud. For a duration of 30 seconds thereafter, the patient views a ball oscillating horizontally in the foreground of the image and reads out numbers as they appear on the ball. The patient cools down after the seventh cycle by walking while listening to self-selected music that facilitates reconnection to the present. Each session is concluded with a post-platform phase which includes debriefing, discussion, reconsolidation, and a mental wellness check/self-care plan. A post-intervention session occurred where an in-depth semi-structured qualitative interview and quantitative questionnaires were conducted. In-depth descriptions of 3MDR have been published elsewhere [9,14]. This protocol described here is slightly modified from the 10-session protocol that is currently used in the Netherlands. 

Further investigation is required regarding the mechanisms by which 3MDR addresses PTSD and components of the intervention that differentiate its efficacy from other therapeutic interventions. One proposed mechanism relates to the manner in which 3MDR addresses emotional regulation (ER) and dysregulation (ED).

### 1.2. Emotional Regulation and Dysregulation

ER is the ability to adaptively respond to negative emotions that arise from threatening or aversive stimuli [15,16]. As proposed by Witte in 1992 [15], ER stems from the inability to directly cope with the presenting threat, resulting in a need to regulate emotions that stem from the experience. According to Gross [16], ER consists of strategies that recognize and respond to negative emotions, such as cognitive reappraisal or redirection of attention from arousing stimuli, as well as inhibition and behavior modulation in response to emotion. These negative emotions include fear, anger, guilt, and shame [17]. ED conversely stems from an inability to perform tasks relating to ER. In PTSD, ED can present itself in the form of both emotional under- and overmodulation [18]. Emotional undermodulation involves hypervigilance, heightened experiences of negative emotions, and poor inhibition and behavioral adaptations in response to negative emotions. This subtype sees decreased frontal lobe activity, particularly in the medial prefrontal cortex (mPFC) and anterior cingulate cortex. On the other hand, emotional overmodulation takes the form of repression, extreme avoidance, inability to perceive one’s emotions, and depersonalization and/or derealization. The overmodulation subtype has excess mPFC and anterior cingulate activity. This study focuses on addressing the emotional undermodulation subtype, and ways in which it is potentially stabilized by 3MDR.

Associated with reduced ER [5,19,20], symptoms of PTSD include negative mood, cognitive deficits, avoidance, and hyperarousal (PTSD diagnostic criteria D, C, and E, respectively). Recent studies suggest that difficulties with ER may exacerbate severity and maintain PTSD symptoms [19,20]. In particular, aspects of ED, such as maladaptive or absent regulatory strategies, avoidance and repression of emotions, and/or unawareness of emotions, have been linked to PTSD symptom severity [21]. Among MMs and veterans, ED can have significant impacts on their ability to function in general and can potentially result in compromised engagement in and maintenance of employment and interpersonal relationships. It can also result in increased risk of harm to self, others, and the mission, and be disruptive to family, social and community functioning, and quality of life. 

There is a crucial need for novel and improved treatment approaches that address all aspects of TR-PTSD among MMs and veterans, including those at adequately address ED as part of PTSD pathology. Currently, 3MDR as a novel therapy is under investigation for its efficacy in addressing combat-related TR-PTSD in military members and veterans. This study aims to examine whether 3MDR is effective at improving ED, and to identify potential neuropsychological mechanisms of 3MDR that may address ER and, consequently, TR-PTSD.

## 2. Materials and Methods

### 2.1. Study Design

This embedded mixed-methods study (quantitative and qualitative) is a sub-study of a larger mixed-methods waitlist control randomized controlled trial (RCT) exploring whether 3MDR is effective at addressing combat-related treatment-resistant PTSD among Canadian MMs and veterans. This sub-study analyzed ER questionnaire data collected using the Difficulties in Emotional Regulation Scale (DERS-18), which is one of several self-reported questionnaires used to assess and monitor symptom changes across the 3MDR intervention. Transcribed data collected during participant sessions, debriefing, and/or postintervention interviews was also used to analyze the functional impact of 3MDR on ED. The study has approval from the University Research Ethics Board (Pro00084466) and the Canadian Armed Forces (CAF) Surgeon General Research Program (E2019-02-250-003-0003).

Two other papers from our group present analyses of certain aspects of the DERS-18 data used in the current study [22,23]. The analyses presented here are distinct from those in the other two papers.

### 2.2. Sample Eligibility and Size

A targeted study sample size of 40 MMs and/or veterans was set for the larger mixed-methods waitlist control RCT to account for a 20 percent dropout rate and allow for power at 32 participants. With four latent variables, for 80% significance at a 5% significance level, the sample size required for this study is 24 (R^2^ = 0.50) [24]. The sample used in the sub-study analyzing ER included data collected from nine participants prior to a pause in data collection due to COVID-19 physical distancing restrictions. 

### 2.3. Inclusion and Exclusion Criteria

3MDR study participants include regular and reserve CAF-MMs and veterans aged 18–60 years under the care of a service provider associated with a Canadian Forces Base, Operational Stress Injury Clinic, or Veterans Affairs Canada. Participants met the Diagnostic and Statistical Manual 5th Edition (DSM5) criteria for PTSD diagnosis [25], had PTSD that was combat-related, had a score of 30 or higher on the Clinician-Administered PTSD Scale for DSM-5 Worst Month version, had been unsuccessful with at least two conventional PTSD treatments, and were stable on their current psychotropic medication for at least four weeks before entering the study. Individuals with comorbidity were included if they satisfied the other inclusion/exclusion criteria. Participants were English-speaking and able to provide informed written consent. A detailed 3MDR protocol has previously been published [14].

### 2.4. Recruitment and Setting

Recruitment was conducted by word of mouth as convenience and snowball sampling. Service providers supporting MMs and veterans, after being introduced to the study via word of mouth and institutional email, informed patients who met the study inclusion and exclusion criteria. Potential participants were provided with a “Permission to Share Contact Information with the Research Team” form by their service provider. Completed forms were forwarded to a researcher who contacted potential participants via phone or email, provided further information about the study, and determined eligibility to participate. Voluntary verbal and written informed consent were obtained from all CAF-MMs and veterans participating in the study. 

The study uses the Computer Assisted Rehabilitation ENvironment (CAREN) system, located in the Glenrose Rehabilitation Hospital in Edmonton, Alberta. Quantitative data, including self-report questionnaires, and qualitative data, including transcripts of participant interviews, were collected preintervention, at each session, and at one week, one month, three months, and six months postintervention. 

### 2.5. Questionnaire

The DERS-18, a shortened yet comparative version of the DERS-36 found to effectively measure ER [21,26], was used in the study. Composed of 18 items, the DERS-18 is divided into six subsections: awareness (lack of emotional awareness), clarity (lack of emotional clarity), goals (hindered goal-related activity), impulse (lack of inhibition), nonacceptance (nonacceptance of negative affective states), and strategies (lack of effective coping strategies). Each subsection is assessed via three focused questions adopted from the DERS-36. Questions are rated on a five-point Likert scale [21], with higher rating indicating greater difficulty with ER. The three questions under the awareness subsection are reverse scored. A high DERS-18 score indicates greater difficulty with ER, and a decrease in DERS-18 score suggests improvement of ED.

### 2.6. Data Collection

Quantitative: Participants were asked to complete a DERS-18 questionnaire prior to starting the 3MDR intervention (T0 baseline) [14]. Following the intervention, DERS-18 data were collected at four follow-up timepoints: one week (T1), one month (T2), three months (T3), and six months (T4) postintervention. Total DERS-18 scores from preintervention T0 were compared to postintervention scores from T2, T3, and T4 to examine change in score following completion of 3MDR. Due to COVID-19 and other limitations, limited T4 scores were collected, and were therefore not included in the analysis. Missing data at varying time points resulted in only five participants having scores at all four pre- and postintervention timepoints. To maximize statistical sensitivity of the analysis, any participant with both pre- and postintervention scores for each of the three pairwise comparisons (T0 vs. T1, T0 vs. T2, T0 vs. T3) were utilized. Nine participants were used to compare T0 and T1 scores (P4, P5, P6, P8, P10, P11, P13, P16, P19), six participants to compare T0 and T2 scores (P4, P5, P8, P10, P13, P19), and six participants to compare T0 and T3 scores (P4, P5, P6, P8, P13, P19). A mean trendline was calculated using data from five participants (P4, P5, P8, P13, P19) from whom data was recorded at all three postintervention timepoints (T3, T4, T5).

Qualitative: Audio and video recordings of data from 3MDR platform sessions, session debriefings, and T1 to T4 follow-up interviews were transcribed and deductively reviewed based on the domains of the DERS-18 for relevant changes in ER. Interview questions (e.g., “How has 3MDR impacted your quality of life, relationships, social integration/engagement, and other meaningful activities or life skills?”) probed changes in ER following 3MDR.

### 2.7. Data Analysis

Changes in DERS-18 scores were analyzed in IBM SPSS using a Wilcoxon signed-ranks test to compare participant preintervention baseline scores and postintervention scores. Participants with preintervention baseline scores (T0) were compared with their respective postintervention scores (T1, T2, T3). The Wilcoxon test is a nonparametric test designed to compare related samples [27]. For each of the three pairwise comparisons, a mean, mean rank, Z score, and *p*-value were collected to analyze for changes in DERS-18 scores. 

Qualitative data was deductively analyzed using descriptive analysis. The rationale behind selecting a deductive descriptive analysis approach was to compare and triangulate the quantitative results found with the DERS-18. Specifically, we sought to explore if participants noticed and/or experienced a tangible change in ER which they would attribute to 3MDR. Qualitative descriptive analysis is a widely cited research tradition and has been identified as important and appropriate for research questions focused on discovering the who, what, and where of events or experiences and gaining insights from informants regarding a less-understood phenomenon [28]. It is also the label of choice when a straight description of a phenomenon is desired, or information is sought to develop and refine questionnaires or interventions [29]. 

A concurrent parallel approach following a data transformation model was utilized in the data analysis process to converge the data for comparing and contrasting the quantitative statistical results with qualitative findings [30,31]. 

## 3. Results

### 3.1. DERS-18 Score 1 Week Postintervention (T0 vs. T1)

Mean participant total DERS-18 score one week following completion of 3MDR intervention (T1) was shown to decrease from baseline preintervention mean participant score (Table 1). The Wilcoxon test generated a statistically significant *p*-value of 0.021 (*n* = 9, Z = −2.314).

### 3.2. DERS-18 Score 1 Month Postintervention (T0 vs. T2)

Mean participant DERS-18 score one month following completion of 3MDR intervention was also shown to decrease from the baseline preintervention mean participant score (Table 1). The conducted Wilcoxon test generated a statistically significant *p*-value of 0.028 (*n* = 6, Z = −2.201). 

### 3.3. DERS-18 Score 3 Month Postintervention (T0 vs. T3)

Mean participant DERS-18 score three months postintervention also showed a decrease from the baseline preintervention mean participant score (Table 1). The Wilcoxon test generated a statistically significant *p*-value of 0.027 (*n* = 6, Z = −2.207).

**Figure 1:**
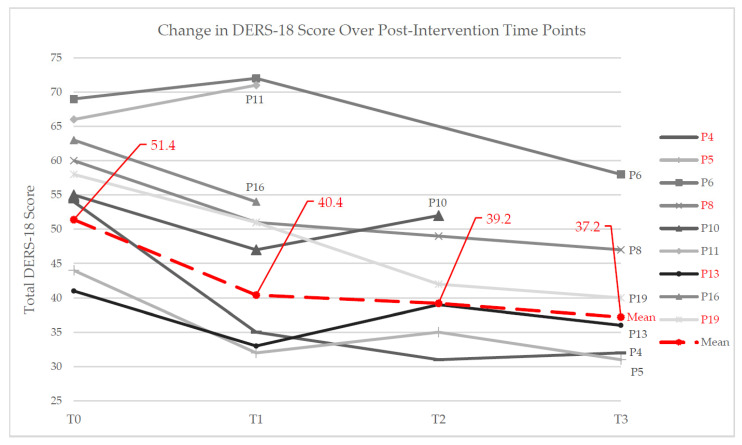
Changes in DERS-18 scores in participants across time. T0 denotes preintervention baseline. T1, T2, and T3 denote postintervention follow-up at 1 week, 1 month, and 3 months. Mean trendline (red dotted line) was calculated using five participants (P4, P5, P8, P13, P19) that had all three postintervention timepoints recorded (T1, T2, T3).

Decreases in DERS-18 score across time are summarized in Table 1 and Figure 1.

### 3.4. Qualitative Changes in Participant Emotional Regulation

Quotes collected from transcribed participant data are displayed in Table 2 and categorized based on relevance to DERS-18 subsections. The qualitative descriptive analysis illustrated that participants expressed a noticeable betterment in all DERS-18 subsections, most significantly in awareness, clarity, nonacceptance, and strategies. Participants expressed that gaining awareness of their emotions, feelings, and sensations; having the correct and appropriate vocabulary to identify these emotions, feelings, and sensations; and then finding strategies (including acceptance) to address ER was central to their treatment and the success of 3MDR.

## 4. Discussion

This study examined whether 3MDR is effective at improving ED among MMs and veterans with TR-PTSD. The results demonstrated a statistically significant decrease in DERS-18 scores (Table 1, Figure 1) for all three pre/postintervention comparisons, with the mean difference in the DERS-18 scores increasing over time. This suggests that ED is improved following 3MDR intervention and signifies potential efficacy of 3MDR in addressing difficulties with ER, as originally hypothesized. The multi-modal and graded, stage-based approach of 3MDR may uniquely account for these positive effects. Qualitative data from the deductive descriptive analysis also indicate noticeable improvement in ER within the subsections of the DERS-18, supporting the quantitative results, with participants reporting noticeable changes in functioning across numerous domains of ER in their day-to-day lives. These positive preliminary results regarding 3MDR’s impact on ER in trauma-affected populations is encouraging. 

Several potential mechanisms of 3MDR may facilitate changes in ER. These include cognitive–motor stimulation, eye movement bilateral stimulation, and comprehensive treatment addressing ER. 

### 4.1. Cognitive–Motor Stimulation

Cognitive-affective and motor systems are functionally interconnected, and subconsciously influence one another [9,32,33]. Body language and motor actions are thought to impact cognitive perception and emotional valence. Neural circuits link sensorimotor regions with diffuse projections [34], such as the cerebellum, striatum, and thalamus, with cognitive-affective areas such as the frontal lobe. The walking motor engagement may act in a bottom-up fashion to guide cognitive and affective appraisal of presented stimuli during 3MDR, potentially controlling ED. These systems are goal-oriented and aim to align stimulus perception and resultant behavior. When there is inconsistency between appraisal and action, the cognitive-affective system, based on the idea of cognitive dissonance, is more inclined to adjust in order to resolve this conflict [35]. Resolution of cognitive dissonance recruits self-mediating and emotionally regulating regions such as the medial prefrontal cortex (mPFC), posterior cingulate cortex, and precuneus [36]. Directionality of movement can also impact appraisal of stimuli to shift more positively or negatively [33]. Therefore, it is hypothesized that the walking approach during 3MDR towards a traumatogenic stimulus may induce a positive shift in appraisal and divergent thinking, reducing avoidance and intensity of negative emotions and perceptions. Cognitive–motor stimulation has previously been studied in relation to ER. Pietrzak et al. [33] addressed ED through controlling breathing patterns, speech tone and content, and posture during CBT. Consequently, stimulating neural networks linking cognitive-affective and sensorimotor function during 3MDR may elevate ER through this proprioceptive feedback.

### 4.2. Eye Movement Bilateral Stimulation

The EMDR working memory task during 3MDR aims to engage neural networks that may both enable the reduction of or disengagement from negative emotions and the fear response and set the stage for reconsolidation of traumatic memories. The working memory task, which has elements of EMDR given its use of eye movements in a sequential manner, is enhanced in 3MDR with the speaking aloud of numbers appearing on an oscillating ball. EMDR is hypothesized to promote neuroplasticity in regulatory regions related to emotional processing [36], such as increased right side intra-hemispheric connectivity. Similarly, EMDR facilitates heightened activity in motor regions involved in the cognitive–motor feedback loop. Areas of the frontal lobe, including the medial and dorsolateral prefrontal cortex [37,38], are stimulated during EMDR, which have been found to be responsible for fear inhibition, fear extinction learning, and ER. These regions have also been implicated to be downregulated in PTSD [38]. The integration of the EMDR task in 3MDR intervention may allow sequential engagement and disengagement from high emotional memories and contribute to a reduction of the valence of negative emotions that result in a need for ER, thereby indirectly addressing ED.

### 4.3. Comprehensive Treatment Addressing Emotional Regulation

Underlying the efficacy of the 3MDR intervention in regulating ED may be the combination of treatment methods (treadmill walking, EMDR, and exposure therapy). Difficulties with ER are highly correlated with severity and maintenance of TR-PTSD [19,20]. 3MDR may facilitate ER in that, layer by layer, dysregulated emotions ranging from intense sadness, grief, loss, or anger, shame, betrayal, or guilt, are addressed in the course of treatment. Affective reconsolidation occurs by correcting and tackling avoidance. Further, experiencing, releasing, naming, and processing raw dysregulated emotions in a safe, predictable context and therapeutic relationship facilitates new meaning-making, memory reconsolidation, and the overlay of newfound memories and experiences over traumatic ones. 

3MDR’s comprehensive and multi-modal approach may affect several neurobiological systems. An immersive, virtual reality-supported, exposure-based therapy, 3MDR may recruit and stimulate prefrontal regions, specifically the mPFC, that are dampened in the emotional undermodulation subtype. Through engaging these areas, 3MDR may facilitate proper inhibition of the amygdala and balance of the amygdalar–prefrontal feedback loop. Further, one of the most significant neural correlates of PTSD is hyperactivation of the amygdala [38,39], which maintains a feedback loop with the regulatory mPFC. The mPFC is related to self-regulation, such as processing and memory of emotions, and sends inhibitory projections to the amygdala, which is responsible for detection of threats, fear conditioning, and emotional intensity [39]. The exposure therapy component in 3MDR involves approaching and engaging with traumatic memories that elicit fear, to effectuate fear extinction learning. Fear extinction requires the mPFC for storage of fear extinction memory to appropriately modulate amygdalar activation [40,41]. Hyperactivity of the amygdala reciprocally inhibits mPFC activation [42,43,44], which can further attenuate mPFC regulation. In PTSD, hyperactivity of the amygdala and decreased white matter prefrontal–amygdalar connectivity is positively correlated with symptom severity [42,43]. Absence of top-down control by the mPFC hinders fear extinction learning in PTSD [43], resulting in heightened arousal, and reduced ER [39,43]. Efficacy of 3MDR in treating difficulties with ER may be derived through extrinsically-induced upregulation of the mPFC to facilitate emotional regulatory processes and reduce intensity and negative appraisal of the traumatic memories (fear extinction). 

ED associated with both PTSD and MI may also be addressed through 3MDR. Exposure to morally injurious experiences, which frequently overlaps with exposure to traumatic experiences associated with combat-related TR-PTSD, is a high-risk factor for the development of MI [4,7], and the comorbidity of MI and PTSD can lead to TR-PTSD. Protopopescu et al. [45] suggest that MI severity is positively correlated with severity of cluster C and D PTSD symptoms (avoidance and negative alterations in cognition and mood; APA, 2013). Although MI is associated with PTSD, it differs in neuropathology [20], and therefore may require alternative treatment approaches. Therefore, addressing ER may be effective in treating MI in addition to PTSD, and may explain 3MDR’s efficacy over other conventional therapeutic approaches. Treatment of MI through 3MDR may further remediate TR-PTSD symptoms, due to the interconnected nature of the two.

### 4.4. Future Research

As a promising, innovative therapeutic intervention that has generated statistically significant outcomes, 3MDR warrants much further study and real-world application with trauma-affected populations. The effect of active participation and engagement of patients by way of walking while simultaneously talking and performing tasks is an area of ongoing investigation. Two RCTs have shown positive results, while a third is underway in the Netherlands and this fourth in Canada. Exploring underlying therapeutic principles and mechanisms involved in 3MDR that individually and collectively address various layers of ED is also an area requiring additional study. To further investigate hypothesized neurobiological mechanisms driving efficacy of 3MDR, and validate the findings from a biological perspective, the study would benefit from implementation of neurohormonal assessment, EEG or other neuroimaging techniques to analyze neuroactivity during, pre-, and postintervention. Continuation of the study and recruitment of further participants will allow more data collection and analysis, enabling greater sample sizes and improving validity of the results. In particular, generating more 6-month postintervention (T4) data would allow for deeper insight on long-term effects of 3MDR on ER. 

### 4.5. Limitations

This study was positioned to assess data in a larger cohort. We experienced some barriers, however, on data collection due to COVID-19 restrictions. As analysis of the presented data sets the stage for new studies, we did not want to delay analysis of the results. We do need to report that, despite these data indicating statistically significant and promising changes in ER following 3MDR, the small sample size limits the validity and generalizability of the results. Ongoing data collection and analysis would allow for determination of stronger conclusions regarding the efficacy of 3MDR on addressing ED. 

## 5. Conclusions

This study reports enhanced ER on a sample of military members and veterans (*n* = 9) with combat-related TR-PTSD following 3MDR, with positive statistically significant preliminary outcomes lasting months following the intervention. It is hypothesized that 3MDR’s comprehensive, multi-modal treatment approach is responsible for the active engagement of suppressed stress regulatory circuits and stimulation of brain circuits (e.g., mPFC, amygdala) pertinent in emotional processing and fear inhibition. These may be underlying mechanisms by which 3MDR addresses cognitive avoidance and ED. Continuing research on this novel intervention’s ability to enhance ER in trauma-affected populations is warranted. The potential impact of 3MDR on ER is not only a promising development for TR-PTSD, but also for other mental health challenges, such as MI, that may contribute to ED. More studies are needed to assess if individuals from diverse other uniformed and civilian populations struggling with trauma-based mental health conditions may benefit equally from the 3MDR intervention.

## Figures and Tables

**Table 1:** Wilcoxon test results. All three comparisons reject the null hypothesis, indicating statistically significant decrease at postintervention from baseline preintervention DERS-18 score.

Post-Intervention Timepoint	1 Week		1 Month		3 Months	
	T0	T1	T0	T2	T0	T3
Mean	56.67	49.56	52.00	41.33	54.33	40.67
df	8		5		5	
*p*-value	0.021		0.028		0.027	
Z score	−2.314		−2.201		−2.207	

**Table 2:** Qualitative narrative analysis: participant quotes describing changes in ER following 3MDR intervention.

	Quotes
Range and layers of emotions addressed by 3MDR	*“[I]t’s a complete 180 in perspective, I am no longer focused on trying to deal with anger and whatever. I am focused on the guilt and shame that caused the anger to begin with. I have gotten to an underlying layer of things … helped me realize it was real, to look at it in a different manner. Shift from anger to shame and guilt which is actually what I had to face. And I had never faced it until now.” (P10)*
Subsection of DERS-18	
Awareness	*“I have noticed [positive] differences. Just being more aware of how I feel. And being able to, you know, once I identify it, I’m able to change the way I–what I’m thinking about. And that’s progress for me because that’s always been a tough one … [to shift out of] an intrusive thought or negative state.” (P5)* *“My emotional range has improved. I don’t avoid stuff anymore, I kind of focus on it, try to deal with it.” (P2)* *“Being more patient. Knowing my reactions. My triggers. What triggers me off, and what sets me off easier. Noticing little things, I used to get so emotionally upset.” (P3)*
Clarity	*“I don’t think I’ve expanded my vocabulary. I think I’ve just learned to put words to feelings … I’m more connected to my emotions and my feelings and my thoughts.” (P5)*
Goals	*“My focus has improved, concentration has improved. Go to work and do paperwork. I still tire quickly when I have to pay attention to details; it does wear me out, but at least I can do it now. Where before I wouldn’t have been able to … improvement is there but it’s not 100%.” (P2)*
Impulse	*“I’m a lot calmer, I’m allowing things to happen without reacting, with the kids and stuff. Stomp, stomp, pound, smash. I’m not letting it get to me, 90% better. I still get irked. You get that twitch. Even my driving is getting better. [Less] reacting to the morons in front, behind and on the side of me.” (P13)*
Nonacceptance	*“In week three, and again in post-week five, was a little bit less of beating myself up, a little bit of saying ‘it’s okay to take care of yourself, it’s okay to put yourself in front sometimes’.” (P11)* *“I used to be very ashamed of [my PTSD]. Very … [Now] everybody I know knows that I suffer from it.” (P6)*
Strategies	*“Usually … I try to deal with [negative thoughts and emotions] in a healthy manner. Spend time with my cat or do photography or just do something different” (P5)* *“[Prior to 3MDR, arguments with wife] would carry on for at least an hour or two. And it would take me 2–3 h to calm down from that. And you know I still get some of those rushes, I guess. But not as bad as they used to be.” (P6)*

## Data Availability

The data presented in this study are available on request from the corresponding author. The data are not publicly available due to its sensitive nature and privacy limitations.
